# Protein complex-based analysis is resistant to the obfuscating consequences of batch effects --- a case study in clinical proteomics

**DOI:** 10.1186/s12864-017-3490-3

**Published:** 2017-03-14

**Authors:** Wilson Wen Bin Goh, Limsoon Wong

**Affiliations:** 1 School of Pharmaceutical Science and Technology, Tianjin University, 92 Weijin Road, Nankai District, Tianjin, 300072 People’s Republic of China; 2 Department of Computer Science, National University of Singapore, 13 Computing Drive, Singapore, 117417 Singapore; 3 Department of Pathology, National University of Singapore, Singapore, Singapore

**Keywords:** Proteomics, Bioinformatics, Principal component analysis, Heterogeneity, Batch effects

## Abstract

**Background:**

In proteomics, batch effects are technical sources of variation that confounds proper analysis, preventing effective deployment in clinical and translational research.

**Results:**

Using simulated and real data, we demonstrate existing batch effect-correction methods do not always eradicate all batch effects. Worse still, they may alter data integrity, and introduce false positives. Moreover, although Principal component analysis (PCA) is commonly used for detecting batch effects. The principal components (PCs) themselves may be used as differential features, from which relevant differential proteins may be effectively traced. Batch effect are removable by identifying PCs highly correlated with batch but not class effect.

However, neither PC-based nor existing batch effect-correction methods address well subtle batch effects, which are difficult to eradicate, and involve data transformation and/or projection which is error-prone. To address this, we introduce the concept of batch-effect resistant methods and demonstrate how such methods incorporating protein complexes are particularly resistant to batch effect without compromising data integrity.

**Conclusions:**

Protein complex-based analyses are powerful, offering unparalleled differential protein-selection reproducibility and high prediction accuracy. We demonstrate for the first time their innate resistance against batch effects, even subtle ones. As complex-based analyses require no prior data transformation (e.g. batch-effect correction), data integrity is protected. Individual checks on top-ranked protein complexes confirm strong association with phenotype classes and not batch. Therefore, the constituent proteins of these complexes are more likely to be clinically relevant.

**Electronic supplementary material:**

The online version of this article (doi:10.1186/s12864-017-3490-3) contains supplementary material, which is available to authorized users.

## Background

The emergence of high-performance protein-extraction procedures (e.g., PCT [1]), brute-force spectra-capture methods (e.g., SWATH [2]), and improved multiplexing technologies [3] has transformed proteomics (the high-throughput expressional study of proteins) from a relatively low-throughput technology to one with critical practical applications in biology.

The application of proteomics on clinical samples (i.e., clinical proteomics) is concerned with unraveling proteome changes associated with disease using actual clinical samples. Typically, two classes of samples---e.g., normal (D) and disease (D*)---are compared against each other. Proteins exhibiting strong inter-class differences are marked as differential and analyzed for relevant functional roles. Statistics provides powerful means for differential protein selection based on the hypothesis-testing framework. This process is commonly referred to as “feature selection” (where a feature is a protein in this instance; see Methods for details on feature selection).

Unfortunately, despite increasing ease in data generation, extracting knowledge from proteomics expression data is difficult [4]. Proper feature selection, if done correctly, should lead directly to drug-target and biomarker identification; but in practice, this is seldom the case [5, 6].

In theory, a strongly differential feature (e.g., a protein) should exhibit strong inter-class differences across samples. However, real samples are intrinsically noisy. This intrinsic noise is random (unstructured) and obfuscates proper feature selection by masking true inter-class differences. The manner in which the samples are prepared contributes towards a second type of variation, which unlike intrinsic random noise, is non-random (structured) and not associated with class effects; i.e., they do not distinguish sample classes D and D* specifically. This second source of variation, where features are more strongly correlated with technical factors (time of experiment, technician, reagent vendor, instrument, etc.) than with sample classes (e.g., D and D*) [7–10], is referred to as batch effects.

It is not straightforward to distinguish batch and class effects: When the former is mild, it may lead to bias during feature selection; but when strong, lead to downright selection of irrelevant proteins that confound and mislead (i.e., false positives) and/or the loss of truly relevant proteins (i.e., false negatives). In other words, batch effects obfuscate analysis. Batch effects are known to be present in genomics assays [7–9]. However, they are a nuisance in proteomics assays, where multiplexing limits impose constraints on the number of samples for concurrent analysis; e.g., analyzing eight samples with the commonly used 4-plex iTRAQ labeling system requires at least two separate experiments performed at different times, or on different instruments.

Despite fairly recent work demonstrating that batch-effect correction may lead to substantial increase in feature-selection sensitivity [11], a systematic exploration of batch effects in proteomics data, and proposal of feasible workarounds, is missing. Reasons include underestimating heterogeneity in practical usage (assuming that class effects dominate variation), unsuitable data (data are already match-paired as ratios and thus, classes cannot be distinguished from each other), and the erroneous belief that normalization eradicates batch effects. Normalization is a data processing technique that adjusts global properties of measurements for individual samples for appropriate comparisons. Examples include z- and quantile-normalization, and mean-scaling. However, normalization cannot eradicate batch effects, as the latter does not affect all variables similarly [10]. In cases where statistical assumptions are violated, normalization may affect data integrity instead.

Batch effects are usually detected via principal component analysis (PCA), where the first two or three principal components (PCs) are plotted for each sample colored by the batch labels, and separation of colors taken as evidence of batch effects [12]. When batch effects are dominant, the first n PCs are expected to be dominated by batch effects, and removal of these PCs may be an alternative yet effective means of batch-effect correction. The remaining PCs---though these have lower contribution towards overall variation---may be dominated by small subsets of variables with good class-discrimination power [13]. Thus, feature selection at the level of PCs---i.e., using PCs, as opposed to proteins, as features---may be a viable batch effect-resistant feature-selection strategy.

Protein complex-based analysis, as a new analytical paradigm, provides a powerful yet stable means of selecting features, at the level of protein complexes, from proteomics data [14–17]. Protein complexes are strongly enriched for biological coherence signal [18], beating any combinations of alternative measurements (expression correlation, GO-term overlaps, etc.) Using protein complex-based analysis, we have successfully recovered missing proteins [17] and overcome consistency issues where patient samples present widely different protein sets [19–21]. Protein complex-based analysis also exhibits unparalleled stability and reproducibility in feature selection [14, 22, 23]. We hypothesize that this superior performance may stem in part, from innate resistance to batch effects.

We address the following gaps in batch effects, and its implications for feature selection in a proteomics setting. First, we propose a simple technique for simulating batch effects in proteomics data, and recommend using it for evaluating feature-selection procedures, as well as checking whether batch effect-correction algorithms are working as intended. Second, while PCA is the *de facto* approach for visualizing presence of batch effects, we investigate its feasibility as a feature-selection technique where features are principal components (PCs) instead of proteins. And finally, as a potential new advantage (which is never reported before), we check whether protein complex-based feature-selection algorithms are truly resistant to batch effects; and if so, whether they may supersede the need for batch effect-correction algorithms.

## Methods

### Simulated data --- D2.2 and D2.2H (Simulated batch effect)

We used part of the D2.2 dataset (301 to 400) from the study of Langley and Mayr as a reference proteomics simulation dataset where differential variables are known a priori [24] (four samples in class D and D* respectively). Quantitation is based on spectral counts.

Class effects and batch effects are inserted randomly, with the increase made in D* samples only. Simulated data with both class and batch effects inserted is referred to as D2.2H, while the original data with only class effects is referred to as D2.2 (Additional file 1).

### Real data --- Renal cancer (RC) (Real batch effect)

The renal cancer (RC) study of Guo et al. [1] comprises a total of 24 SWATH runs originating from six pairs of non-tumorous and tumorous clear-cell renal cell carcinoma (ccRCC) tissues, in two batches, rep1 and rep2 (Additional file 1).

### Batch effect-correction methods

For batch-effect correction, we used quantile normalization and linear-scaling as generic approaches (Additional file 1). Quantile normalization and linear-scaling are not explicitly batch effect-correction methods. So, we also used COMBAT on D2.2H to remove batch effects, and evaluate performance recovery against the original D2.2 (where no batch effects are introduced but class effects are). COMBAT is a well-known batch effect-correction approach and employs empirical Bayes frameworks for adjusting data for batch effects. It is reported to be robust to outliers in small sample sizes (<25) while maintaining comparable performance to existing methods for large samples [25].

### Statistical feature-selection methods

Four classes of feature-selection methods are tested to see whether they are robust against batch effects (i.e., they do not select features that are associated with batch). The standard Single-Protein *t*-test (SP) [26] and Hypergeometric Enrichment (HE) [4] test are the most commonly used comparative analysis methods. We have also included two variants of rank-based network algorithms (RBNAs)---viz. SubNETs (SNET) [27] and Fuzzy-SubNETs (FSNET) [23]---which were demonstrated to be highly stable and reliable [14] (Additional file 1).

On real data, HE, SNET and FSNET are tested using CORUM complexes [28] as their protein complex-based feature vector [16, 17, 29]. The performance of these feature-selection methods are evaluated on precision and recall (Additional file 1).

On simulated data (D2.2 and D2.2H, without and with simulated batch effects respectively), these same methods are evaluated based on simulated complexes (pseudo-complexes). In simulated data, the differential proteins are known a priori. We use these to create true-positive pseudo-complexes. To achieve this, a Euclidean distance is first determined for all differential protein pairs across all samples. These are then clustered via Ward’s linkage. Differential proteins are reordered such that those with similar expression pattern (across samples) are adjacent to each other. This reordered list is then split at regular intervals to generate 101 true-positive pseudo-complexes. An equal number of non-significant proteins is randomly selected, reordered based on expressional correlation, and then split to generate an equal number of true-negative pseudo-complexes.

We may alter the “purity” of the true-positive pseudo-complexes by reducing the proportion of differential proteins within them. In practice, we seldom observe all complex members being differentially expressed simultaneously (which also renders it too easy for detection). Purity, therefore, is the proportion of differential proteins within each true-positive pseudo-complex. At 100% purity, simulated complexes are comprised solely of significant proteins; at 75% purity, 25% of the constituent significant proteins are randomly replaced with non-significant ones; and so on. Reducing purity permits evaluation of the robustness and sensitivity of the complex-based analysis methods. Purity is tested at three levels: 100, 75 and 50%.

The true-positive and true-negative pseudo-complexes are combined into a single vector. Evaluation is based on the F-score.

## Results and discussion

### Batch effects cannot be completely eradicated via batch effect-correction algorithms

**Fig. 1:**
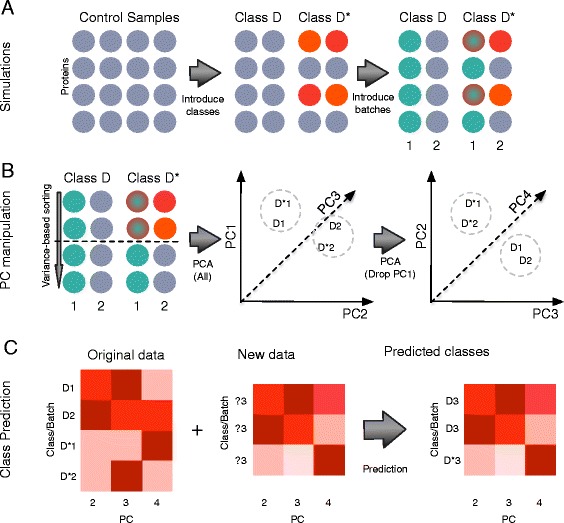
**a** Simulations: From single-class data, samples are split randomly into two classes D and D*. Class effects are randomly inserted into proteins in D*. This is followed by insertion of batch effects. **b** PC manipulation: Proteins are sorted based on variance followed by a cutoff. The retained expression data is analyzed via principal component analysis (PCA). The first principal component (PC1) corresponds strongly to batch effects and may be removed. **c** Manipulation of PCs for clustering and class prediction. When combined with data of unknown labels, class labels can be predicted based on co-clustering with samples of known labels

Our method simulates batch effects in the following manner (Fig. 1a): In the first dimension, class-effect sizes are inserted based on the method of Langley and Mayr to distinguish classes D and D* [24]. Class-effect sizes are sampled randomly from five possibilities and inserted into randomly selected variables on samples belonging to class D*, constituting 20% of all variables. This is repeated to generate 100 random datasets (D2.2). In the second dimension, for each of the 100 datasets, batch effects are inserted over all variables. Here, batch effects are simulated by taking half the members in D and D* to be batch 1, and the remaining half as batch 2. Batch effects are not evenly applied across all variables, and thus cannot be eradicated via simple normalization [10]. To simulate batch effects unevenly, heterogeneity/batch-effect sizes, like class-effect sizes, are also sampled randomly from the same five possibilities. However, we make the assumption that batch effects influence all variables in a sample, and thus expect batch effects to account for a majority of variation. These 100 simulated datasets, D2.2.301H to D2.2.400H are available for download from Additional file 2.

**Fig. 2:**
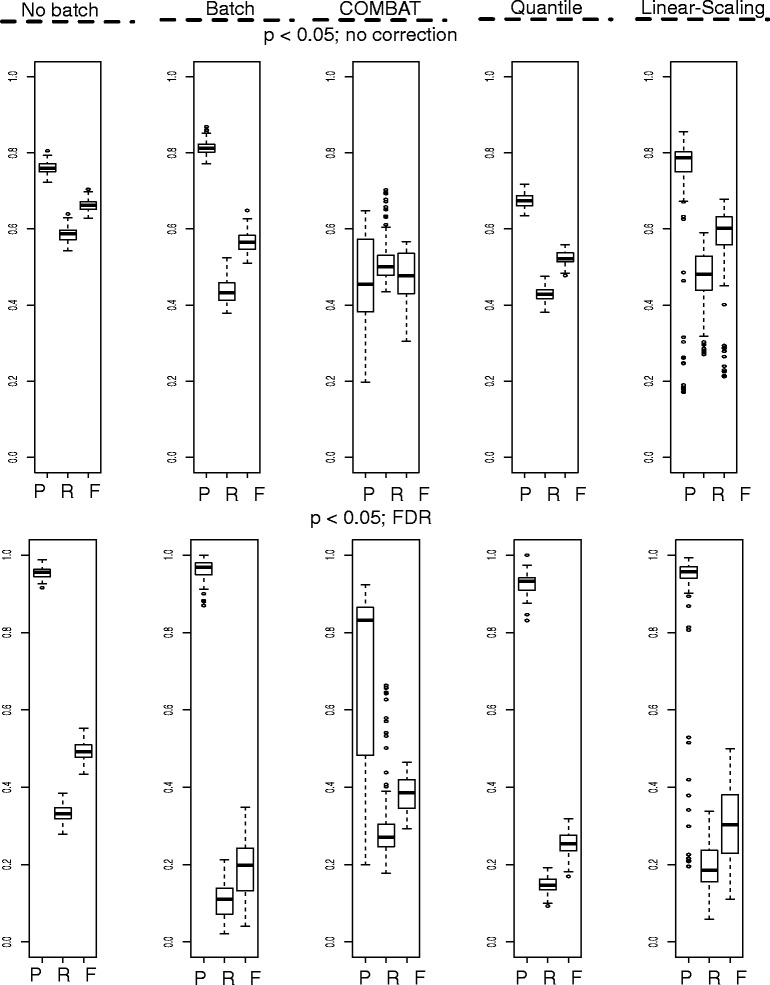
Batch effect-correction does not work optimally in practice. Top row: Feature selection without multiple-test correction. Bottom row: Feature selection with FDR correction. First column shows base performance with only class effects (No batch). Second column shows performance with batch effects incorporated. Third, fourth and fifth columns show recovery using various batch effect-correction methods, (COMBAT, quantile normalization and linear-scaling). Feature-selection test used here is the two-sample *t*-test (*Abbreviations*: P, Precision; R, Recall; F, F-score)

To understand how batch effects affect feature selection, we compare precision, recall and F-score based on the standard *t*-test, with and without multiple-test correction based on the Benjamini-Hochberg False Discovery Rate (FDR) (Fig. 2). The first two columns in Fig. 2 reveal that incorporation of batch effects increases the variability of performance metrics, with particular impact on recall, and reduces overall performance (viz. F-score). While we expect different feature-selection methods, aside from the *t*-test, may respond differently to batch effects, it is useful to incorporate heterogeneity/noise simulations during performance evaluation.

The commonly used batch effect-correction method, COMBAT [25], only partially recovers original test performance (without batch effects). Therefore, it does not completely eradicate heterogeneity. Additionally, while it improves overall performance, it also tends to reduce precision, incorporating false positives into the selected feature set. This is cause for concern during selection of features for experimental validation. In the non-FDR corrected scenario, COMBAT also does not perform better than conventional data normalization methods, e.g., quantile normalization and linear-scaling (Fig. 2). However, when test requirements are more stringent given the 5% FDR cutoff, then it is clear that COMBAT provides considerable advantage (over both quantile normalization and linear-scaling).

In spite of the simplicity of these simulations, it is noteworthy that batch effect-removal (and normalization) methods are not a panacea. We cannot declare COMBAT is inferior, but rather, we will never know if batch effects have been effectively removed from real data, particularly when the data happens not to fit COMBAT’s assumptions well. Thus, a naïve reliance on batch effect-correction algorithms, without conducting further downstream checks for remnant batch effects (if possible), may potentially worsen analytical outcome.

### A relook at principal component analysis for detecting and removing batch effects

Principal component analysis (PCA) yields linear combinations of each variable’s contribution to variance, but evidently not all variables are equally interesting or relevant (which necessitates feature selection in the first place): We may say the features that changed the most, i.e., exhibited the most variation, are likely more impactful (contributing strongly to class or batch effects, or both). Although we may not know this first-up for every feature, we can still reduce the feature set size via variance-based pre-selection. Here, a cutoff is introduced to include only the top 20% proteins (ranked by variance) and used in PCA (Fig. 1b).

**Fig. 3:**
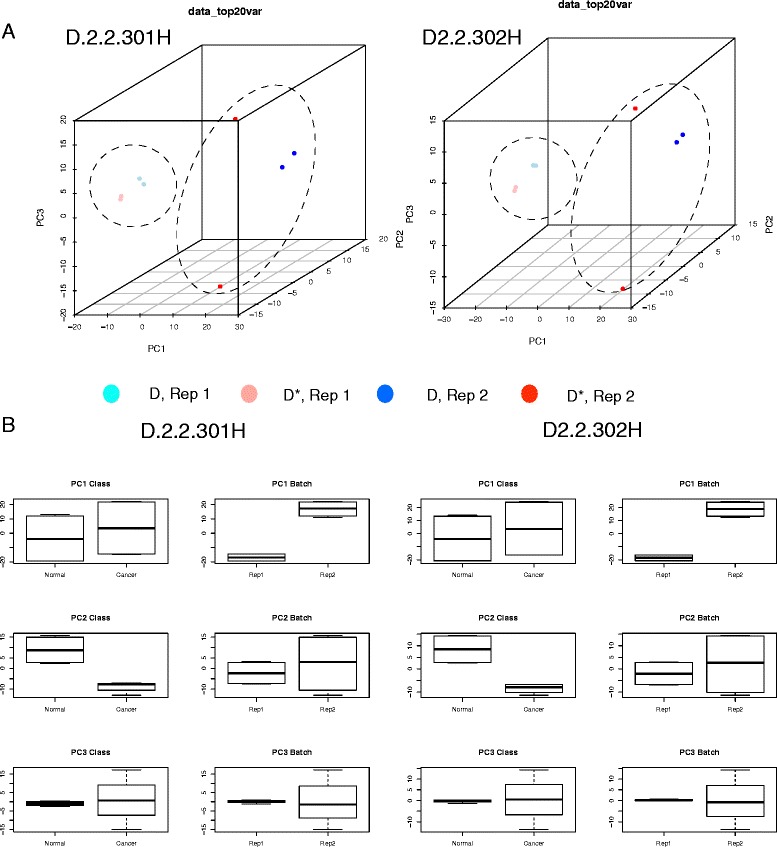
**a** 3D-Principal Component Scatterplot Analysis: Batch effects dominate in the simulated data. Shown are two examples D2.2.301H and D2.2.302H. **b** PC-paired boxplots: Each PC may be split by class and batch, and compared against each other as side-by-side boxplots. This allows easy evaluation of the contribution of class and batch effects to each PC

As stated earlier, PCA is commonly used for visualizing batch effects. A simple way to do this is to label samples by classes (D and D*) and batches (rep 1 and 2), and diagrammatize these as scatterplots across the first 2 or 3 principal components (PCs). In Fig. 3a, the PCs are based on the top 20% proteins (ranked by variance) and evidently, this is sufficient for detecting batch effects. It is therefore unnecessary to use all variables (Additional file 3). However, it is unclear whether this is sufficient for detecting class effects.

Scatterplots, being rough visual guides, do not reveal well the contributions of class and batch effects to each PC. In our opinion, paired boxplots (splitting each PC by class and batch) are more informative and, here, it is evident that PC1 and PC2 correspond to batch and class effects respectively (Fig. 3b).

**Fig. 4:**
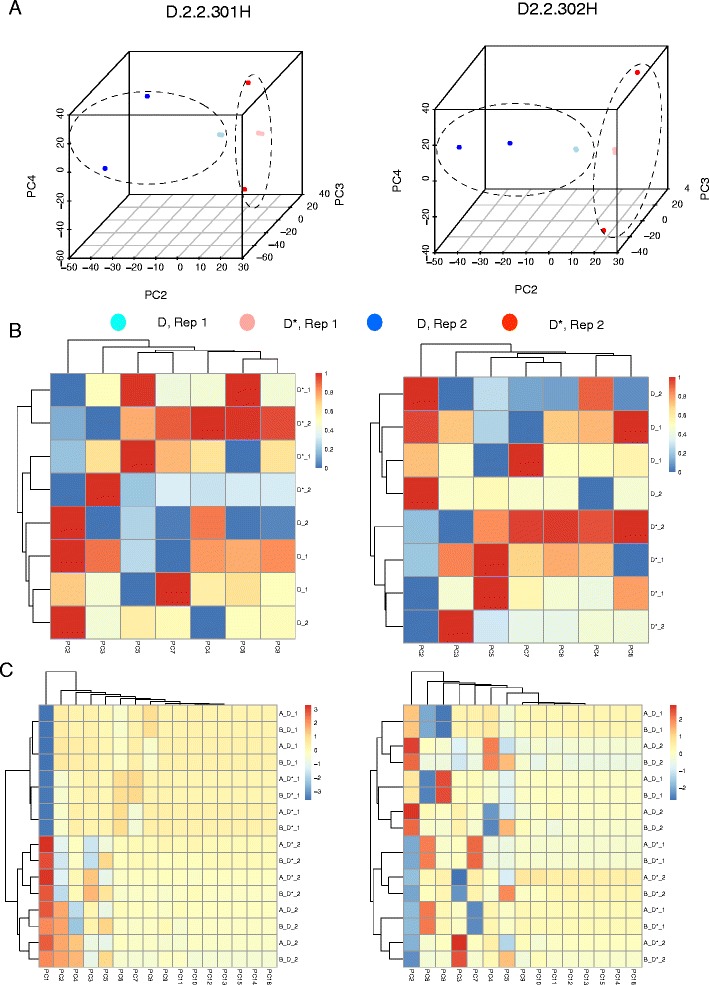
**a** 3D-Principal Component Analysis: Class effects dominate in the simulated data, given the removal of PC1 (Two examples are shown: D2.2.301 and D2.2.302). **b** Heatmaps and hierarchical clustering (HCL): The remaining PCs may also be used as individual variables for clustering, and provide strong discrimination between classes D and D*. **c** Combining two datasets with different batch effects: Datasets A and B have the same differential feature set but different batch effects. Combining these followed by analysis of all principal components (PC) shows batch effects dominate. However, removal of PC1 perfectly recovers class-effect discrimination without having to perform any feature selection (Notation: A/B_D/D*_1/2 refers to the dataset, class and batches respectively)

When a batch effect is observed, it is common practice to apply a batch effect-removal or –correction method (e.g., COMBAT [9]). However, this does not necessarily work well in practice. Moreover, if the data does not fit the correction method’s assumptions, it may lead to false positives. Instead, we may opt for a more direct strategy by simply removing the first PC (Fig. 1b), and deploying the remaining PCs as features for analysis. When PC1 contributes strongly to batch effects, its removal should allow class effects to become the dominating source of variation (Fig. 4a).

Using two examples (D2.2. 301H and 302H), we show that removal of the first PC (PC1) allows samples to cluster based on classes rather than batch (Fig. 4a). A caveat is that removal of PC1 works here primarily because it is strongly correlated with batch effects; i.e., batch effects account for the majority of variance in the data. On real data, it may necessitate the removal of several other PCs that are correlated with batch effects. Moreover, if incompletely eradicated or inseparable from class effects, batch effects may resurface in subsequent PCs (after PC1) during analysis (See section “Variable-selection methods with resistance to batch/heterogeneity effects”).

Suppose that removal of the first n PCs results in good class separation in PCA, it may be possible to use the remaining PCs for feature selection and non-projection-based clustering techniques, e.g., hierarchical clustering and k-means. This may seem counter-intuitive, as during standard analysis involving PCA, it is common to keep just the top n PCs accounting for the majority of variation. But not all variation is attributable towards class effects (even if it is large). Moreover, PCs with large same-sign coefficients tend to represent non-class effect properties correlated with the variables; e.g., Tsuchiya et al. demonstrated that, for their dataset, PC1 is linearly correlated with the magnitude of average gene expression [30]. On the other hand, subsequent PCs with lower contribution towards overall variation may be dominated by small subsets of variables with good class-discrimination power [13]. Thus, instead of discarding the lower ranked PCs, it is more reasonable to remove the top PCs that are non-correlated with class effects. We find that subsequent PCs do correlate strongly with sample classes D and D* (Fig. 4a), and may be used as variables for clustering (Fig. 4b).

A PC-based feature-selection approach is viable and allows relevant proteins to be retraced. This is executed by first identifying the PC of interest, and selecting proteins that contribute exclusively and strongly to it [13]. As a simple test, we look at two scenarios using D2.2.301H and D2.2.302H. In the first scenario, proteins strongly associated with PC1 (without PC1 removal) are selected. We term this “PC1” (see Table 1). In the second scenario, we removed the original PC1, and looked at proteins strongly associated with the new PC1 (the new PC1 is computed on the reduced data where those proteins strongly associated with the original PC1 are removed). This is termed “–PC1” (see Table 1). Since the original PC1 is strongly associated with batch effects, its associated proteins are therefore less relevant, and we expect that performing feature selection on these using the *t*-test, and evaluating the corresponding precision, recall and F-score should fare worse than those proteins associated with the new PC1. We find the results following PC1 removal are similar to batch effect-correction algorithms, with concomitant increase in recall following removal of the first principal component (Table 1). As a parallel test, we calculate a t-statistic for each protein under consideration, rank them from the highest to lowest, and keep only the top n proteins, where *n* = same number of proteins associated with –PC1 (termed “TT/-PC1” in Table 1). Interestingly, these do worse (lower precision and recall), and moreover, have limited overlaps with the –PC1 selected features.

**Table 1:** Effects on precision and recall for D2.2.301H and D2.2.302H before and following removal of proteins with heavy loadings on the first principal component (PC1 and − PC1 respectively)

	Precision	Recall	F-score	Jaccard
301H (PC1)	1.00	0.30	0.46	
301H (-PC1)	1.00	0.35	0.52	
301H (TT/-PC1)	0.65	0.23	0.34	0.48
302H (PC1)	0.94	0.32	0.48	
302H (-PC1)	0.93	0.43	0.59	
302H (TT/-PC1)	0.72	0.30	0.42	0.61

TT refers to the standard *t*-test while TT/-PC1 are the top n-ranked TT-features (restricted to the number of features in –PC1). The Jaccard coefficient indicates limited overlaps amongst the top TT-features associated with –PC1 features

This procedure---viz. rank proteins by variance, perform PCA using the top 20%, discard PCs that are strongly correlated with technical variables, and perform e.g., clustering using the remaining PCs---may be used for class prediction on new batches with unknown class labels. A schematic is provided in Fig. 1c. And, to test this, two different sets of batch effects are inserted into D2.2.301, the first is (20, 50, 80, 100 and 200%), and the second is (10%, 30%, 50%, 70%, 90%) (Fig. 4c), and the data combined.

Expectedly, clustering based on all PCs show that batch effects dominate. However, removal of PC1 recovers perfect class discrimination. This suggests that even when combining several datasets with different batch effects, removal of PC1 retains class effects, and permits class prediction (Fig. 1c). Moreover, if we have multiple datasets of the same disease, properly dealing with batch effects makes it possible to pool these datasets for analysis. This is useful when larger sample sizes are needed for ad hoc analysis.

### Using variance-based variable pre-selection and principal component manipulation to tackle real batch effects

To evaluate how the procedure above is applicable towards real data, we consider the renal cancer study of Guo et al., which contains two technical replicates (i.e., two batches, rep1 and rep2)[1]. This data, RC, has been carefully processed; and batch effects appear contained (Additional file 4).

**Fig. 5:**
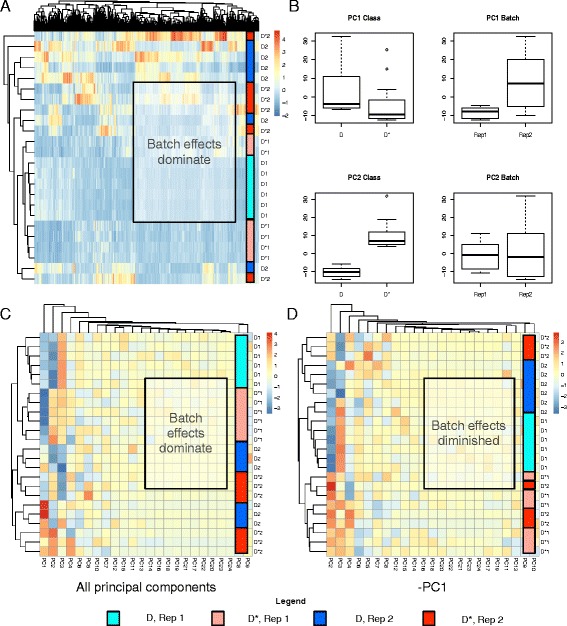
**a** Hierarchical Clustering (HCL) based on protein expression: Batch effects dominate here, as samples separate more strongly based on batch than by class. No variable selection was performed prior. **b** Principal Components (PCs) 1 and 2 stratified by class and batch: Although PC1 is still strongly associated with batch; there is also some association with class effects. In contrast, PC2 is strongly associated with class rather than with batch effects. **c** HCL based on all PCs: In this case, it is clear that batch effects dominate. **d** HCL following PC1 removal: Removal of PC1 appears to eradicate a large proportion of the batch effects. But this is incomplete, suggesting that there may still be ambient batch effects remaining

We insert batch effects into RC rep2 (in the same manner as D2.2H) (Fig. 5a). However, PC1 does not purely contain variability from batch effects but also some signal from class effects (Fig. 5b). As with D2.2H, PC projection also reveals dominance of batch effects (Fig. 5c) that may be controlled via removal of PC1 (Fig. 5d). However, it appears some remnant batch effect still persists (Some rep2 D and D* samples still cluster together).

### Feature-selection methods with resistance to batch/heterogeneity effects

There are remnant batch effects in RC (Fig. 5d) that are difficult to eradicate, and may lead to bias during subsequent feature selection. When batch effects are strong, then removal of the first few PCs is a useful direct strategy, especially if information on batch and other potential confounding factors are not known a priori (i.e., we cannot systematically eliminate non-class relevant variation) and batch effect-correction methods cannot be effectively deployed. On the other hand, it is not always straightforward to interpret the PCs and extract the proteins relevant for class effects. PC-based removal and batch-effect correction also may not be able to remove subtle batch effects.

As an additional note of caution, batch effect-removal approaches---including the procedure described above---may at times be overkill: These corrections may unintentionally eliminate true biological heterogeneity amongst samples (i.e., disease subpopulations), which is informative (e.g., identifying personalized signatures for determining therapy) and should not be discarded from the data in the first place. Unfortunately, batch-effect and subpopulations are not easy to tell apart [16]. And if we run the PC-removal or other batch effect-correction methods, subpopulation information is irrevocably lost. On the other hand, high heterogeneity in the form of multiple subpopulations can make analysis very challenging, particularly in cancer proteomics [31].

One way forward is to incorporate robust data normalization methods and biological context (e.g., networks and protein complexes) directly into feature-selection approaches [29]. Recently, we expound on the advantages of protein complexes as suitable biological context in improving data analysis. Unlike analysis at the level of proteins as features, the use of protein complexes as features, leads to improve stability and reproducibility [14, 15, 20, 21, 32, 33].

We are curious if the high performance of protein complex-based methods belonging to the family known as Rank-Based Network Analysis (RBNAs) exhibit superior performance (high feature-selection reproducibility and cross-validation prediction power) due to innate resistance to batch effects [14, 23, 27]. There are several reasons why we think RBNAs may be robust against batch effects: Its score function uses rank-based discretization instead of exact values, which is robust against various biases, e.g., test-set bias [34]. Use of biological context (e.g., networks and complexes) increases biological signal over signal from other spurious correlations (e.g., batch) as only signal from same-complex members are summated. We already know that use of protein complexes increases power, and we believe the signal amplification is phenotypically relevant [35]. Previous tests have already demonstrated that complex-based features are specifically predictive for phenotype classes and that false-positive rates are low. However, a specific investigation into batch-effect resistance has not been done. Hence, we test two members: SubNets (SNET) [27] and Fuzzy-SNET (FSNET) [23].

To test whether RBNAs are effective in overcoming batch effects, as opposed to simply eliminating them, we performed two sets of tests; the first on simulated data (based on D2.2 and D2.2H) as a proof-of-concept and the second on real data (using RC). Besides SNET [27] and FSNET [23] (two representative RBNA methods), we also include the standard single-protein *t*-test (SP) [26, 36] and the hypergeometric enrichment test (HE) [4]. SP is a control based on the standard univariate *t*-test at the level of individual proteins. HE is an over-representation-based technique meant to determine if the differential proteins are significantly enriched in some protein complex based on the hypergeometric test; i.e., it uses the same protein complexes, but not the same statistical test as the RBNAs.

**Fig. 6:**
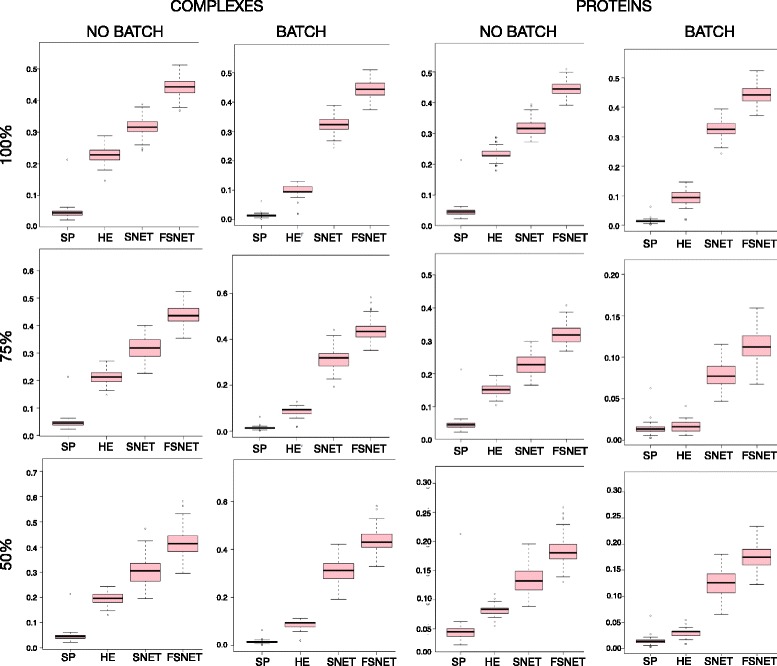
Complex-based methods are robust against batch effects (simulations): Distribution of F-scores for SP, HE, SNET and FSNET in simulated data where batch effects are absent or present based on the simulated complexes (pseudo-complexes) and individual proteins. Note that for SP, the F-scores shown are always based on individual proteins (*Abbreviations*: Single-Protein *t*-test, SP; Hypergeometric Enrichment, HE; SubNET, SNET; Fuzzy SNET; FSNET)

Using D2.2 (no batch effects) and D2.2H (simulated batch effects), we compare the F-scores across three purity levels (proportion of differential proteins within the pseudo-complexes), at both complex and individual protein levels (Fig. 6). We observe that the F-score for the protein-based *t*-test (SP) is very low to begin with, but progressively worsens when batch effects are introduced. The hypergeometric enrichment (HE) pipeline uses the same pseudo-complexes as the RBNAs, but also appears to be sensitive to batch effects (especially at the complex level). The RBNAs, SNET and FSNET, are resistant to batch effects. At the complex level, there is almost no difference in F-scores regardless of purity. This suggests that the RBNAs are robust not only against the batch effects, but also decreasing differential signal as purity decreases. This finding is also corroborated at the level of individual proteins as well.

Our findings are a positive indication that the RBNAs are highly robust against weaker differential signal and batch effects. Additionally, given HE’s poor performance, we assert that use of complexes alone is insufficient; the statistical test setup is also critical. This result is important as it is the first, to the best of our knowledge, to demonstrate that complex-based feature-selection approaches are resilient against batch effects.

However, there are caveats: Firstly, these batch effects are simulated, and unreflective of true batch effects. Secondly, these pseudo-complexes may not be good approximations of true protein complexes. Although we cannot test real data directly (the real differential proteins are not known a priori), we may still evaluate these methods (SP, HE, SNET and FSNET) on real data.

**Fig. 7:**
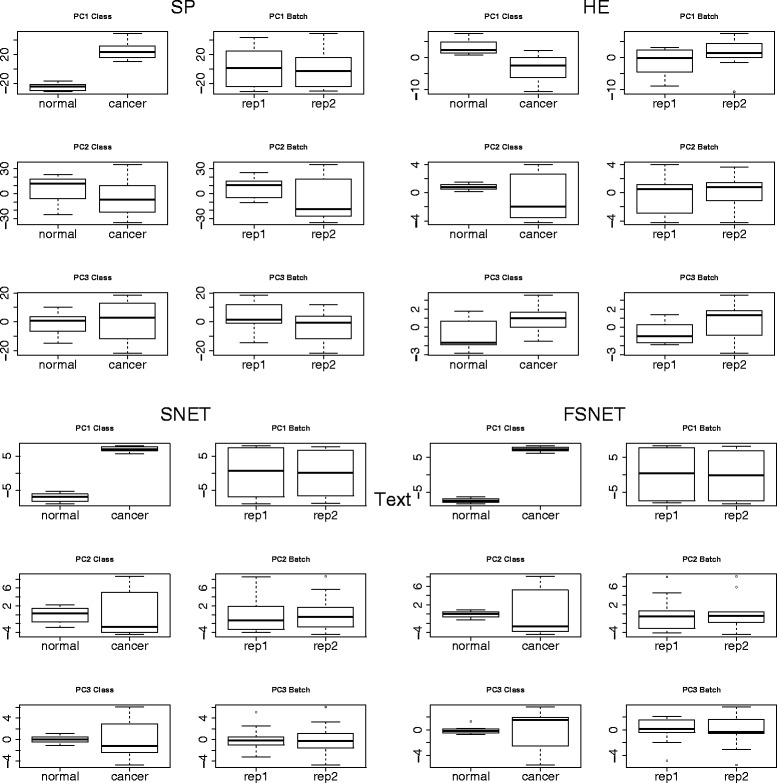
PC side-by-side boxplots stratified by class and batch tested on real data:, Rank-based network algorithms SNET and FSNET are against batch effects, and only seems to capture variation stemming from class effects (*Abbreviations*: Single-Protein *t*-test, SP; Hypergeometric Enrichment, HE; SubNET, SNET; Fuzzy SNET; FSNET)

Using the original RC (where ambient batch effects persist), we perform PCA on significant features selected by the four feature-selection methods. For the first 3 PCs, side-by-side boxplots stratified by class and batch (Fig. 7) demonstrates that RBNAs (SNET and FSNET) are very powerful, and appear to capture variation stemming only from class effects. This is in contrast to SP, where batch effects still persist in PC2. HE is commonly used as a post-hoc test following SP. It also takes advantage of the same set of protein complexes as SNET and FSNET; but clearly, this is insufficient (batch effects are relegated to PC3): The method used for statistical testing also matters.

This finding is critical: Since RBNAs are robust against batch effects, this obviates the need for performing data transformations (e.g., PCA or batch-effect correction). This also means that if subpopulations do exist in the data, this information is retained. It should be noted that dealing with subpopulations is difficult and outside the scope of this work, although we may also use complexes for detailed in-depth study of subpopulations [16].

While RBNAs are evidently powerful against batch effects, especially against subtle ones that cannot be easily removed via removal of the first n PCs or via batch effect-correction algorithms, they are not perfect solutions. E.g. methods such as FSNET weigh each protein in a protein complex by the fraction of subjects (in the relevant class) where the protein is highly ranked. This fraction may be unstable from subsample to subsample, particularly in the presence of hidden subpopulations. This may reduce class-specific signals, making it difficult to identify good-quality and relevant features.

### Downstream considerations post-analysis

Discarding batch effect-laden PCs is a transformation that provides a transformed dataset with much reduced batch effects. It helps identify strong class discriminatory features. Yet, at the same time, new batches are not directly comparable to this transformed dataset. So it is difficult to extract directly clinical guideline/thresholds for future diagnostic use. For example, as a simple diagnostic tool, one wants a protein X such that: If X’s abundance is above a threshold y, then the patient is sick. But, in the presence of batch effects, different thresholds are needed for different batches. On the other hand, once the good features are found, one may apply more reliable technologies---i.e., ones that are less susceptible to batch effects---to measure only those features specifically.

**Fig. 8:**
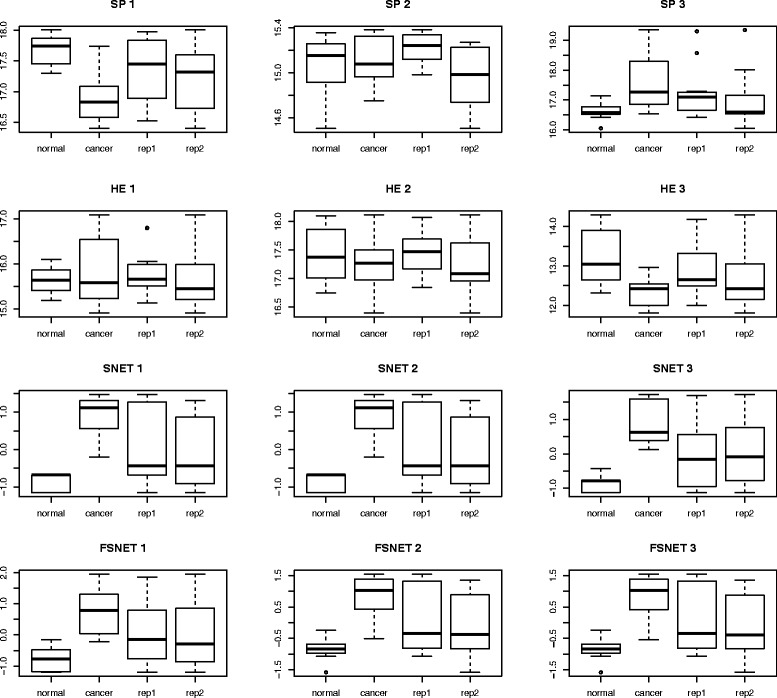
Expression or network score-based (y-axis) side-by-side boxplots stratified by class and batch for top three variables: Rank-based network algorithms, SNET and FSNET, can capture the class effects while robust against batch effects. Both class and batch variability is present in the top variables selected by SP and HE (*Abbreviations*: Single Protein *t*-test, SP; Hypergeometric Enrichment, HE; SubNETs, SNET; Fuzzy SubNETs; FSNET)

To test this empirically, the analysis in Fig. 7 is repeated. We plot the boxplots based on log-normalized expression of significant proteins for SP and HE, and scores of significant subnets for SNET and FSNET, for the top three features (Fig. 8). For SP and HE, both class and batch variation are detected amongst the top three features. So it is reasonable to conclude that where these approaches are concerned, it is difficult to identify class discriminatory signal. The top three features selected by SNET and FSNET (i.e., subnets) capture class effects better, and appear robust against batch effects. Thus, we expect that use of these features for diagnostics will yield better results.

## Conclusions

The impact of batch effects in proteomics cannot be understated, and has key implications in clinical and translational research. We have shown that batch effect-correction algorithms are not a panacea, and that the corrected data may be erroneous. Moreover, with the development of any novel feature-selection approach, it is worthwhile to test their robustness against simulated batch effects.

We have illustrated that side-by-side barplots are better for visually detecting batch effects than the standard PCA scatterplot-based representation format. Moreover, the PCs themselves may be used as features, which may also be effectively traced back to relevant differential proteins. This is also a viable strategy complementary to batch effect-correction methods.

Unfortunately, subtle batch effects cannot be easily removed or detected, and can lead to bias in the analysis of real data. Moreover, data transformation may lead to the loss of valuable subpopulation information. We confirm that one of the reasons complex-based algorithms like the RBNAs are successful is because they have innate resistance against batch effects. This resistance stems from amplification of phenotypic-relevant signal from same-complex members and rank-based discretization of expression values (increasing the signal from high confidence proteins while removing noise from low confidence proteins). As RBNAs require no prior data transformations, the integrity of the data is preserved (including subpopulation information). Finally individual check on the top features selected by RBNAs confirms that they are strongly associated with class and not batch effects. This means that features selected in this manner are more likely to be clinically useful.

## Additional files

Additional file 1: Descriptions of supplementary methods [1, 4, 14, 17, 24, 26, 37, 38]. (DOCX 96 kb)

Additional file 2: Simulated data D2.2H. (ZIP 3730 kb)

Additional file 3: 3D-Principal Components Analysis (PCA) scatterplots for all variables in samples D2.2.301H and D.2.2.302H. B: 3D-Principal Components Analysis (PCA) scatterplots for top 20% variables (ranked by variance) in samples D2.2.301H and D.2.2.302H. (DOCX 192 kb)

Additional file 4: Batch-effects in RC appears to be limited (left: All variables; right: Top 20% variables ranked by variance). (DOCX 125 kb)
