# Supplementary material for: Protein complex-based analysis is resistant to the obfuscating consequences of batch effects --- a case study in clinical proteomics
Source: BMC Genomics. 2017 Mar 14;18(Suppl 2):142. doi: 10.1186/s12864-017-3490-3 (PMC5374662; doi:10.1186/s12864-017-3490-3)
Supplement: Supplementary file 1 — Descriptions of supplementary methods [1, 4, 14, 17, 24, 26, 37, 38]. (DOCX 96 kb) [file 12864_2017_3490_MOESM1_ESM.docx]

# Additional Methods

### Quantile normalization

Quantile normalization is a technique for making several different distributions (corresponding to samples in different datasets or batches) identical in statistical properties. It involves first constructing a data matrix where the columns are samples, and rows correspond to variables (proteins). Then, order each column (regardless of batch) by values. Then, average across rows and substitute the values of each row by this average. Finally, in each column, reorder the averaged values back into the original order.

### Linear-scaling

In linear-scaling, for each sample, find the value X_min_, and the maximum value, X_max_. For all variables in the sample, subtract by X_min_ and divide by the delta of X_max_ – X_min_. This conversation will bound the data values between 0 and 1. Linear-scaling shifts all data points by a fixed magnitude but does not change the data distribution.

### Single-protein t-test (SP)

The two-sample t-test for selection of single proteins is performed by calculating a t-statistic (*T_p_*) and its corresponding nominal p-value for each protein *p* by comparing the expression scores between classes *C_1_* and *C_2_*, with the assumption of unequal variance between the two classes [26]:

$$T_{p}=\frac{\bar{x}_{1}-\bar{x}_{2}}{\sqrt{\frac{s_{1}^{2}}{n_{1}}+\frac{s_{2}^{2}}{n_{2}}}}$$

where $\bar{x}_{j}$ is the mean expression level of the protein *p*, *s_j_* is the standard deviation and *n_j_* is the sample size, in class *C_j_*.

### Hypergeometric enrichment test (HE)

HE is a traditional form of subnet-based feature selection that is frequently used and consists of two steps [4]. First, differential proteins are identified using the two-sample t-test (see above). This is followed by a hypergeometric test where given a total of *N* proteins (with *B* of these belonging to a complex) and *n* test-set proteins (i.e., differential), the exact probability *P* that *b* or more proteins from the test set are associated by chance with the complex is given by:

$$P\left( X \geq b \right)=\sum_{i=b}^{min(n,B)} \frac{\binom{n}{i}\binom{N-n}{B-i}}{\binom{N}{B}}$$

The sum $P\left( X \geq b \right)$ provides the p-value of the hypergeometric test.

### SubNETs (SNET) and Fuzzy SubNETs (FSNET)

SNET and FSNET are examples of rank-based network algorithms [14]. They differ from HE in terms of data processing and subnet test statistic calculation. For SNET, given a protein *g_i_* and a tissue *p_k_*, let *fs*(*g_i_*,*p_k_*) = 1, if the protein *g_i_* is among the top alpha percent (default = 10%) most-abundant proteins in the tissue *p_k_*; and = 0 otherwise.

Given a protein *g_i_* and a class of tissues *C_j_*, let

$$\beta\left( g_{i},C_{j} \right)=\sum_{p_{k} \in C_{j}} \frac{fs(g_{i},p_{k})}{|C_{j}|}$$

That is, $\beta\left( g_{i},C_{j} \right)$ is the proportion of tissues in *C_j_* that have *g_i_* among their top alpha percent most-abundant proteins.

Let *score*(*S*,*p_k_*,*C_j_*) be the score of a protein complex *S* and a tissue *p_k_* weighted based on the class *C_j_*. It is defined as:

$$score(S,p_{k},C_{j})=\sum_{g_{i} \in S} fs\left( g_{i},p_{k} \right)*\beta\left( g_{i},C_{j} \right)$$

The function $f_{SNET}\left( S,X,Y,C_{j} \right)$ for some complex *S* is a t-statistic defined as:

$$f_{SNET}\left( S,X,Y,C_{j} \right)=\frac{mean\left( S,X,C_{j} \right)-mean(S,Y,C_{j})}{\sqrt{\frac{var(S,X,C_{j})}{|X|}+\frac{var(S,Y,C_{j})}{|Y|}}}$$

where *mean*(*S*,#,*C_j_*) and *var* (*S*,#,*C_j_*) are respectively the mean and variance of the list of scores { *score*(*S*,*pk*,*C_j_*) | *p_k_* is a tissue in # }.

The complex *S* is considered differential (weighted based on *C_j_*) in *X* but not in *Y* if *f_SNET_*(*S*,*X*,*Y*,*C_j_*) is at the largest 5% extreme of the Student t-distribution, with degrees of freedom determined by the Welch-Satterwaite equation.

Given two classes *C_1_* and *C_2_*, the set of significant protein complexes returned by SNET is the union of {*S* | *f_SNET_*(*S*,*C_1_*,*C_2_*,*C_1_*) is significant} and {*S* | *f_SNET_*(*S*,*C_2_*,*C_1_*,*C_2_*) is significant}, the former being complexes that are significantly consistently highly abundant in *C_1_* but not *C_2_*, the latter being complexes that are significantly consistently highly abundant in *C_2_* but not *C_1_*.

FSNET is identical to SNET, except in one regard:

For FSNET, the definition of the function *fs*(*g_i_*,*p_k_*) is replaced such that *fs*(*g_i_*,*p_k_*) is assigned a value between 1 and 0 as follows: *fs*(*g_i_*,*p_k_*) is assigned the value 1 if *g_i_* is among the top alpha1 percent (default = 10%) of the most-abundant proteins in *p_k_*. It is assigned the value 0 if *g_i_* is not among the top alpha2 percent (default = 20%) most-abundant proteins in *p_k_*. The range between alpha1 percent and alpha2 percent is divided into *n* equal-sized bins (default *n*=4), and *fs*(*g_i_*,*p_k_*) is assigned the value 0.8, 0.6, 0.4, or 0.2 depending on which bin *g_i_* falls into in *p_k_*.

A test statistic *f_FSNET_* is then defined analogously to *f_SNET_*. Given two classes *C_1_* and *C_2_*, the set of significant complexes returned by FSNET is the union of {*S* | *f_FSNET_*(*S*,*C_1_*,*C_2_*,*C_1_*) is significant} and {*S* | *f_FSNET_*(*S*,*C_2_*,*C_1_*,*C_2_*) is significant}.

## Simulated data --- D2.2 (Simulated batch effect)

We used part of the D2.2 dataset (301 to 400) from the study of Langley and Mayr as a reference proteomics simulation dataset where differential variables are known *a priori* [24] (4 samples in class D and D* respectively). Quantitation is based on spectral counts.

D2.2.301 to D2.2.400 comprise 100 simulated datasets each with 20% randomly generated significant variables. This corresponds to 710 significant proteins. The class-effect sizes of these 20% differential variables are sampled from one out of five possibilities or p (20%, 50%, 80%, 100% and 200%), and the increase is made in D*. This is expressed as:

$${SC}_{i,j}'={SC}_{i,j} * (1+p)$$

where SC_i,j_ and SC_i,j_’ are respectively the original and simulated spectral count from the j^th^ sample of protein i.

To simulate batch effects, two control and two test samples are assigned to rep 1, and the remaining samples rep 2. All proteins in rep 2 samples are randomly assigned a batch effect, also drawn from one out of five possibilities or p (20%, 50%, 80%, 100% and 200%), and added onto the spectral count (as above).

## Real data --- Renal cancer (RC) (Real batch effect)

In Guo *et al*. [1], all SWATH maps are analyzed using OpenSWATH [37] against a spectral library containing 49,959 reference spectra for 41,542 proteotypic peptides from 4,624 reviewed SwissProt proteins [1]. The library is compiled via library search of spectra captured in DDA mode (linking spectra mass-to-charge and retention time coordinates to a library peptide). Protein isoforms and protein groups are excluded from this analysis. Proteins are quantified via spectral count, which is the total number of MS/MS spectra acquired for peptides from a given protein.

## Network-based feature vector using natural protein complexes

As HE, SNET and FSNET are network-based algorithms, they require comparison of the proteomics data against a feature vector comprised of subnets, which may be predicted from reference networks, or taken from data repositories of known/validated protein complexes. The gold standard for protein complex data is the CORUM database, which contains manually annotated protein complexes from mammalian organisms [38]. In earlier studies, real complexes are demonstrated to be superior to predicted subnets from protein-interaction networks [17]; so we use these.

## Precision, Recall and the F-score

For a given variable-selection method (where variables are proteins), we may evaluate its performance on simulated data where true positives are known *a priori*, using precision and recall:

$$Precision= \frac{TP}{TP+FP};Recall= \frac{TP}{TP+FN}$$

where TP, FP and FN are the true positives, false positives and false negatives respectively. Precision and recall are both important. To simplify analysis, they may be combined based on the harmonic mean. This is also referred as the F-score (*F_S_*):

$$F_{s}=2*\frac{Precision * Recall}{Precision+Recall}$$
