# Supplementary material for: Protein complex-based analysis is resistant to the obfuscating consequences of batch effects --- a case study in clinical proteomics
Source: BMC Genomics. 2017 Mar 14;18(Suppl 2):142. doi: 10.1186/s12864-017-3490-3 (PMC5374662; doi:10.1186/s12864-017-3490-3)
Supplement: Supplementary file 3 — 3D-Principal Components Analysis (PCA) scatterplots for all variables in samples D2.2.301H and D.2.2.302H. B: 3D-Principal Components Analysis (PCA) scatterplots for top 20% variables (ranked by variance) in samples D2.2.301H and D.2.2.302H. (DOCX 192 kb) [file 12864_2017_3490_MOESM3_ESM.docx]

Additional file 3: 3D-Principal Components Analysis (PCA) scatterplots for all variables in samples D2.2.301H and D.2.2.302H. B: 3D-Principal Components Analysis (PCA) scatterplots for top 20% variables (ranked by variance) in samples D2.2.301H and D.2.2.302H.
