# Supplementary material for: Protein complex-based analysis is resistant to the obfuscating consequences of batch effects --- a case study in clinical proteomics
Source: BMC Genomics. 2017 Mar 14;18(Suppl 2):142. doi: 10.1186/s12864-017-3490-3 (PMC5374662; doi:10.1186/s12864-017-3490-3)
Supplement: Supplementary file 4 — Batch-effects in RC appears to be limited (left: All variables; right: Top 20% variables ranked by variance). (DOCX 125 kb) [file 12864_2017_3490_MOESM4_ESM.docx]

Additional Figure 4 Batch-effects in RC appears to be limited (left: All variables; right: Top 20% variables ranked by variance).
